# T185. DIFFERENTIAL ACTIVITY OF TRANSCRIBED ENHANCERS IN THE PREFRONTAL CORTEX OF 592 CASES WITH SCHIZOPHRENIA AND CONTROLS

**DOI:** 10.1093/schbul/sby016.461

**Published:** 2018-04-01

**Authors:** Panos Roussos

**Affiliations:** Mount Sinai School of Medicine

## Abstract

**Background:**

Transcription at enhancers is a widespread phenomenon, which produces so-called enhancer RNA (eRNA) and occurs in an activity dependent manner. The role of eRNA and its utility in exploring disease-associated changes in enhancer function and the downstream coding transcripts that they regulate is however not well established. We here used transcriptomic and epigenomic data to interrogate the relationship of eRNA transcription to disease status and how genetic variants alter enhancer transcriptional activity in the human brain.

**Methods:**

We combined RNA-seq data from 537 post mortem brain samples from the CommonMind Consortium with cap analysis of gene expression and enhancer identification, using the assay for transposase-accessible chromatin followed by sequencing.

**Results:**

We find 118 differentially transcribed eRNAs in schizophrenia and identify schizophrenia-associated gene/eRNA co-expression modules. Perturbations of a key module are associated with the polygenic risk scores. Further, genetic variants affecting expression of 927 enhancers, which we refer to as enhancer expression quantitative loci or eeQTLs, are identified. Enhancer expression patterns are consistent across studies, including differentially expressed eRNAs and eeQTLs. Combining eeQTLs with a genome-wide association study of schizophrenia identifies a genetic variant that alters enhancer function and expression of its target gene, GOLPH3L.

**Discussion:**

Here, we expanded the scope of the CommonMind Consortium to interrogate enhancer function in schizophrenia, to examine how genetic variation affects enhancers, and to evaluate specific effects on enhancer and gene expression for previously identified schizophrenia risk variants. Our novel approach to analyzing enhancer transcription is adaptable to other large-scale, non-poly-A depleted, RNA-seq studies.

